# A chemical with proven clinical safety rescues Down-syndrome-related phenotypes in through DYRK1A inhibition

**DOI:** 10.1242/dmm.025668

**Published:** 2016-08-01

**Authors:** Hyeongki Kim, Kyu-Sun Lee, Ae-Kyeong Kim, Miri Choi, Kwangman Choi, Mingu Kang, Seung-Wook Chi, Min-Sung Lee, Jeong-Soo Lee, So-Young Lee, Woo-Joo Song, Kweon Yu, Sungchan Cho

**Affiliations:** 1 Anticancer Agent Research Center, Korea Research Institute of Bioscience and Biotechnology, Cheongju, Chungbuk 28115, Republic of Korea; 2 Department of Biomolecular Science, University of Science and Technology, Daejeon 34113, Republic of Korea; 3 Neurophysiology Research Group, Hazard Monitoring BioNano Research Center, Korea Research Institute of Bioscience and Biotechnology, Deajeon 34141, Republic of Korea; 4 Department of Functional Genomics, University of Science and Technology, Daejeon 34113, Republic of Korea; 5 Disease Target Structure Research Center, Korea Research Institute of Bioscience and Biotechnology, Daejeon 34141, Republic of Korea; 6 International Cooperation Office, Ministry of Food & Drug Safety, Cheongju, Chungbuk 28159, Republic of Korea; 7 Department of Biochemistry and Molecular Biology, Neurodegeneration Control Research Center, School of Medicine, Kyung Hee University, Seoul 02447, Republic of Korea

**Keywords:** Down syndrome, Alzheimer's disease, DYRK1A, CX-4945, Tau hyperphosphorylation

## Abstract

DYRK1A is important in neuronal development and function, and its excessive activity is considered a significant pathogenic factor in Down syndrome and Alzheimer's disease. Thus, inhibition of DYRK1A has been suggested to be a new strategy to modify the disease. Very few compounds, however, have been reported to act as inhibitors, and their potential clinical uses require further evaluation. Here, we newly identify CX-4945, the safety of which has been already proven in the clinical setting, as a potent inhibitor of DYRK1A that acts in an ATP-competitive manner. The inhibitory potency of CX-4945 on DYRK1A (IC_50_=6.8 nM) *in vitro* was higher than that of harmine, INDY or proINDY, which are well-known potent inhibitors of DYRK1A. CX-4945 effectively reverses the aberrant phosphorylation of Tau, amyloid precursor protein (APP) and presenilin 1 (PS1) in mammalian cells. To our surprise, feeding with CX-4945 significantly restored the neurological and phenotypic defects induced by the overexpression of *minibrain*, an ortholog of human DYRK1A, in the *Drosophila* model. Moreover, oral administration of CX-4945 acutely suppressed Tau hyperphosphorylation in the hippocampus of DYRK1A-overexpressing mice. Our research results demonstrate that CX-4945 is a potent DYRK1A inhibitor and also suggest that it has therapeutic potential for DYRK1A-associated diseases.

## Abstract (precis)

**Editors' choice:** *In vivo* validation of a potent DYRK1A inhibitor, with proven clinical safety, using Down-syndrome- and Alzheimer's-disease-like models.

## INTRODUCTION

Dual-specificity tyrosine phosphorylation-regulated kinase 1A (DYRK1A) is a serine/threonine kinase important to brain development. DYRK1A plays a variety of functional roles within the adult central nervous system (Tejedor and Hammerle, 2011). The significance of DYRK1A has been recently highlighted by the discovery of its contribution to Down syndrome (DS) pathogenesis.

DS is the most common genetic disorder, with an incidence of 1 in 800 live births, and is caused by a complete or partial trisomy of chromosome 21. DS is characterized by various symptoms, including mental retardation and congenital heart defects, as well as by defects in immune and endocrine systems (Becker and Joost, 1999). These featured phenotypes are closely associated with the overexpression or hyperactivity of many genes that can be mapped within the Down syndrome critical region (DSCR) on chromosome 21 (Guimera et al., 1999; Wiseman et al., 2009). In particular, mental retardation, which is a characteristic symptom of DS, is thought to be related primarily to the *DYRK1A* gene in the DSCR (Smith and Rubin, 1997). Many studies using different lines of transgenic mice have shown that the additional expression of DYRK1A in a normal mouse, which mimics trisomy in human DS, is sufficient to cause abnormalities in learning and memory as well as brain structure, strongly suggesting a central function for DYRK1A in the mental retardation associated with DS (Ahn et al., 2006; Altafaj et al., 2001). Moreover, mice with lowered DYRK1A expression show phenotypic effects similar to those in mice overexpressing DYRK1A, indicating that DYRK1A activity is tightly controlled during normal brain development and that a dosage imbalance in DYRK1A expression affects brain structure and function (Arque et al., 2008; Benavides-Piccione et al., 2005; Fotaki et al., 2002, 2004).

Intriguingly, increased DYRK1A activity has been also reported in various brain compartments in subjects that suffer from Alzheimer's disease (AD), a representative neurodegenerative disease (Ferrer et al., 2005; Tiraboschi et al., 2004). At the neuropathological level, DS and AD share several features that are characterized by the presence of amyloid plaques and neurofibrillary tangles (NFTs), the formation of which is affected by the aberrant phosphorylation of Tau (for NFTs), as well as of amyloid precursor protein (APP) and presenilin 1 (PS1) (for amyloid plaques) (Johnson and Hartigan, 1999; Tiraboschi et al., 2004). Moreover, it has been reported that DYRK1A directly phosphorylates Tau, APP and PS1 (Ryoo et al., 2008, 2007; Ryu et al., 2010). These observations provide a plausible link between DS and AD that could explain the early onset of AD-like symptoms in the majority of people with DS and further indicate that DYRK1A could be a promising therapeutic target for treating diseases such as DS and AD that involve DYRK1A overexpression or hyperactivity.

Despite substantial efforts to develop potent and selective inhibitors of DYRK1A, only a few are currently available, and their potential clinical use remains to be tested further (Smith et al., 2012). Extensive evaluations of the most promising DYRK1A inhibitors that have been developed to date suggest that their therapeutic application might still be limited by pharmacological side effects.

Here, we report CX-4945 as a novel inhibitor of DYRK1A with a high potency. Its strong inhibitory effect on DYRK1A has been extensively confirmed *in vitro*, in mammalian cells and even in living organisms. Moreover, we pharmacologically validated the use of CX-4945 *in vivo* in model organisms by observing the effective rescue of neurological and phenotypic defects in a DS-like *Drosophila* model, and the significant suppression of Tau phosphorylation in the hippocampus of DS-like mice. As a potent inhibitor of DYRK1A with proven safety in clinical trials, CX-4945 will be a valuable tool in DYRK1A-related basic research and in the development of therapeutic drugs for DYRK1A-associated diseases, such as DS and AD.

## RESULTS

### Identification of CX-4945 as a novel inhibitor of DYRK1A

**Fig. 1.**
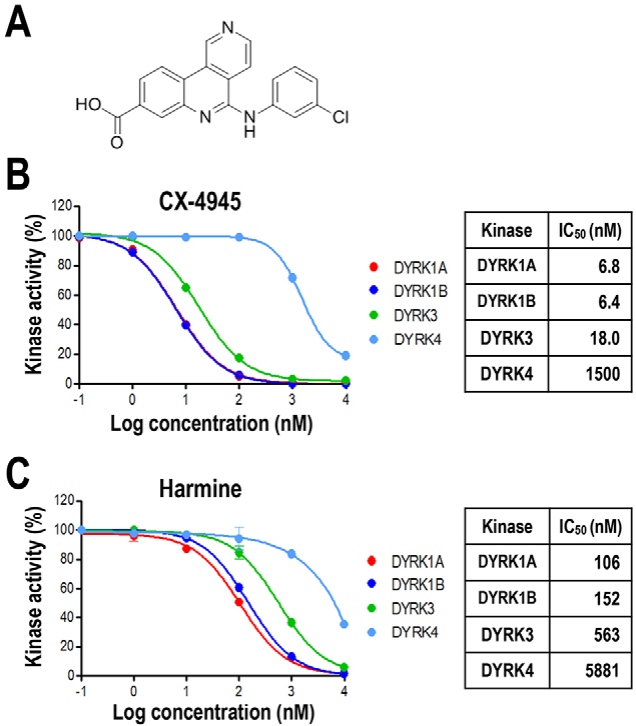
**CX-4945 is a potent inhibitor of DYRK1A *in vitro*.** (A) Diagram of the chemical structure of CX-4945. (B) Potent inhibition of DYRKs by CX-4945 was observed in *in vitro* kinase assays conducted by Life Technologies using recombinant human DYRK1A, DYRK1B, DYRK3 and DYRK4 proteins. The mean values and standard deviation (s.d.; error bars) were determined from two independent assays. The IC_50_ values were determined using PRISM software. (C) Inhibition of DYRKs activity by harmine was measured as described for CX-4945 in B.

Our recent research has demonstrated that CX-4945, a previously well-characterized inhibitor of casein kinase 2 (CK2) and a molecule currently in phase 1b and phase 2 clinical trials for cancer treatment, is a potent inhibitor (IC_50_=∼3-10 nM) of Cdc2-like kinases (Clks), which regulate alternative splicing (Kim et al., 2014; Siddiqui-Jain et al., 2010) (Fig. 1A). Intriguingly, many small-molecule inhibitors of Clks (TG-003, KH-CB19 and Leucettine L41) inhibit DYRKs with potencies similar to those for their inhibition of Clks (Debdab et al., 2011; Fedorov et al., 2011; Mott et al., 2009). This could be explained by the phylogenetic similarity between DYRKs and Clks (Aranda et al., 2011; Kannan and Neuwald, 2004). In fact, along with CK2 and Clks, DYRKs are classified as part of the CMGC superfamily of proline- or arginine-directed serine/threonine kinases. Therefore, we tested whether CX-4945 also has an inhibitory effect on DYRKs using *in vitro* kinase assays with human recombinant kinases and a synthetic peptide substrate (see *In vitro* kinase assays in Materials and Methods). We found that CX-4945 potently inhibited the activity of all DYRK-family proteins (IC_50_=6.8, 6.4, 18 and 1500 nM for DYRK1A, DYRK1B, DYRK3 and DYRK4, respectively; Fig. 1B). Among them DYRK1A and DYRK1B were most strongly affected by CX-4945, and its potency was much higher (about 20-fold) than that of harmine, a potent DYRK inhibitor that is widely used (Adayev et al., 2011) (Fig. 1C). Among the DYRK-family proteins, DYRK1A is a considerable pathological factor for DS; therefore, further studies were focused on the DYRK1A protein.

### CX-4945 inhibits DYRK1A in an ATP-competitive manner

**Fig. 2.**
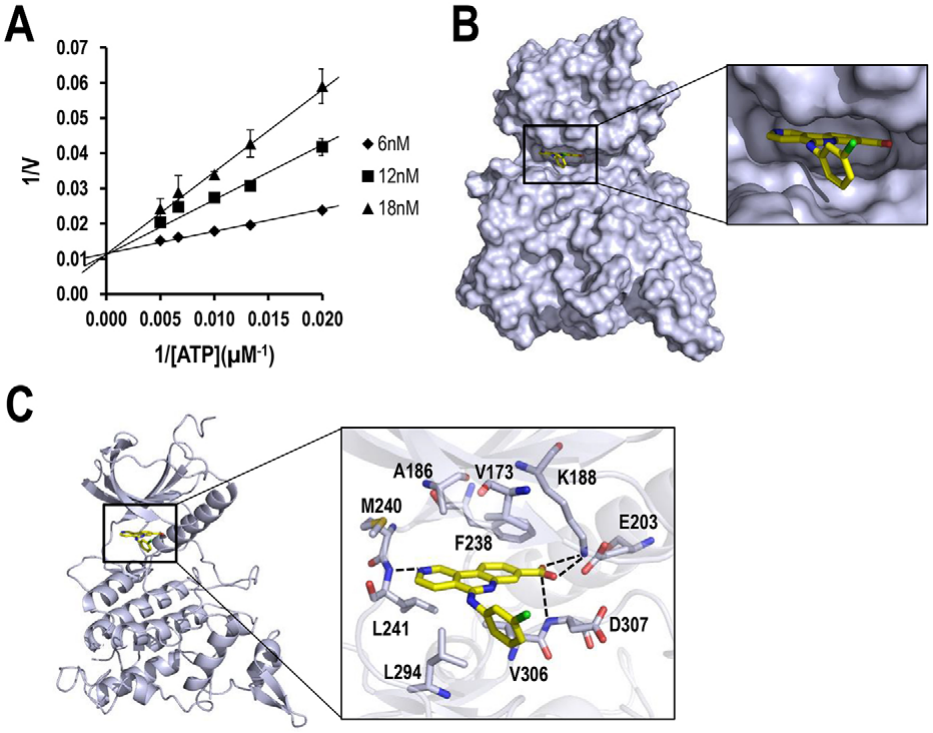
**ATP-competitive inhibition of DYRK1A by CX-4945.** (A) Lineweaver–Burk plots for ATP in the presence of CX-4945 at various concentrations. Data are mean±s.d. (B) Structural model of DYRK1A complexed with CX-4945, which is predicted to bind to the ATP-binding site of DYRK1A. (C) The predicted binding mode of CX-4945 to the ATP-binding pocket of DYRK1A. Amino acids in the ATP-binding pocket of DYRK1A are denoted by gray sticks, and CX-4945 is indicated by yellow sticks. Hydrogen bonds are denoted as dotted black lines, and oxygen, nitrogen and chlorine atoms are indicated as red, blue and green sticks, respectively.

Previously, CX-4945 has been found to inhibit the activities of CK2 and Clks through binding to the ATP-binding pocket and competing with ATP (Ferguson et al., 2011; Kim et al., 2014). Therefore, to examine whether CX-4945 also acts in this manner, we measured DYRK1A kinase activity with various combinations of ATP and CX-4945 concentrations *in vitro*. As a result, CX-4945 inhibited DYRK1A in an ATP-competitive manner, as expected (Fig. 2A). Moreover, molecular docking studies using the CDOCKER program in Discovery Studio software were performed to gain deeper insights into the orientation of CX-4945 in the ATP-binding pocket of DYRK1A, and also into the interaction between DYRK1A and CX-4945 at the molecular level. Consequently, we found that CX-4945 fits snugly into the ATP-binding pocket of DYRK1A (Fig. 2B) and makes extensive hydrophobic interactions with residues V173, A186, F238, M240, L241, L294 and V306 in DYRK1A. Additionally, four hydrogen bonds were predicted to be made between CX-4945 and the adjacent residues (K188, L241 and D307) (Fig. 2C). In particular, the hydrogen bonds between the carboxyl group of CX-4945 and the side-chain nitrogen of K188 in DYRK1A appear to be crucial for this binding, given that a similar hydrogen bond has also been observed for the binding of CX-4945 to human protein kinase CK2α (Ferguson et al., 2011) (Fig. 2C). Overall, these results strongly support the notion that CX-4945 directly inhibits DYRK1A through ATP competition.

### CX-4945 is a potent inhibitor of DYRK1A

**Fig. 3.**
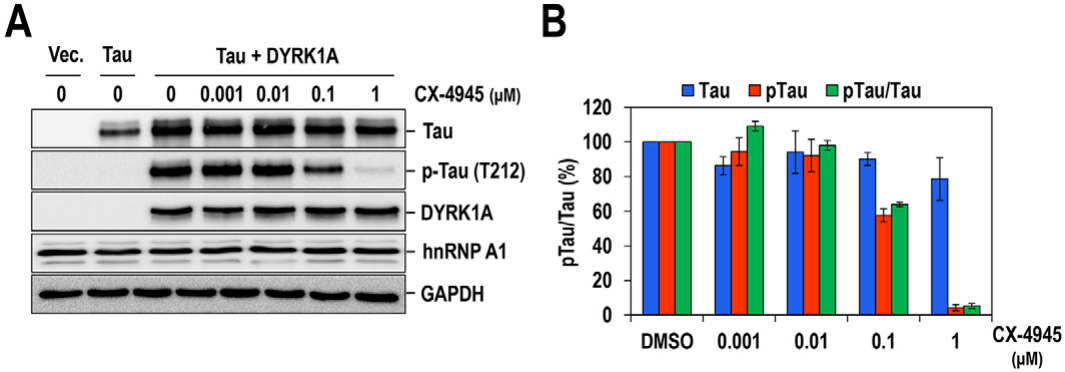
**CX-4945 potently inhibits DYRK1A-mediated Tau phosphorylation in mammalian cells.** (A) Tau phosphorylation by DYRK1A in mammalian cells is inhibited by CX-4945. 293T cells were transfected with plasmids expressing Tau and DYRK1A, and then treated with the indicated doses of CX-4945 for 6 h. Total cell extracts were prepared and subjected to western blotting with anti-Tau, anti-phosphorylated-Tau (at T212, p-Tau) and anti-DYRK1A antibodies. The hnRNP A1 and GAPDH proteins were also monitored as controls. Western blotting was performed twice, and representative data are presented. (B) The phosphorylated and total Tau proteins from western blotting were quantified, and the amount of protein relative to that in the DMSO-treated samples are presented. The mean±s.d. was determined from two independent experiments. pTau/Tau, the ratio of phosphorylated Tau to total Tau. Vec, empty vector.

**Fig. 4.**
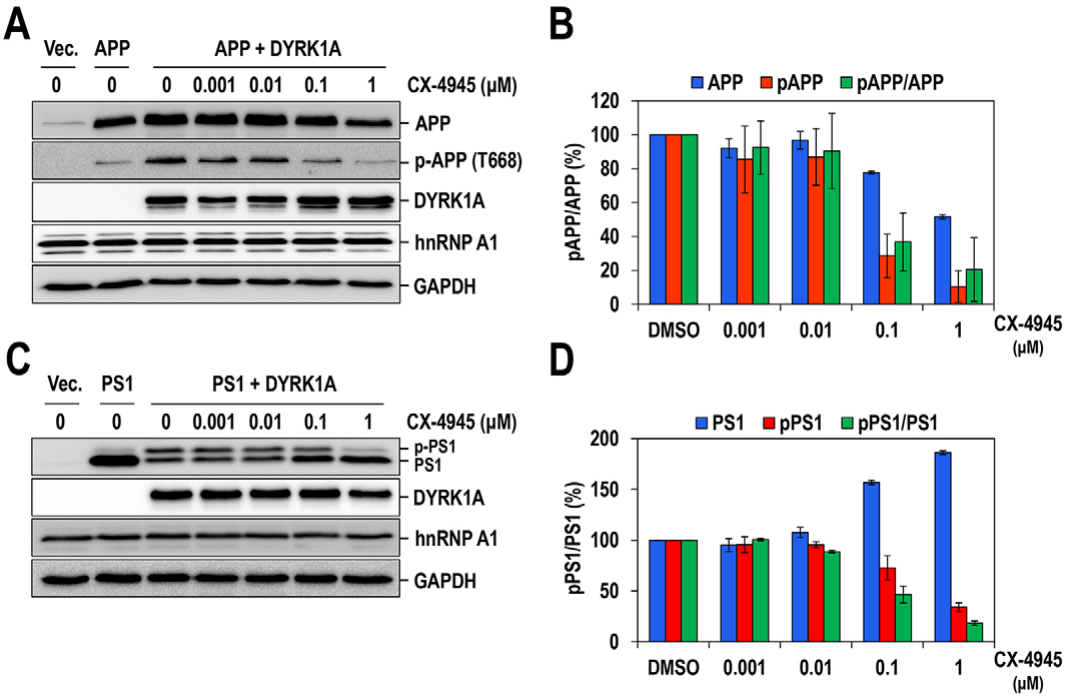
**CX-4945 inhibits DYRK1A-mediated APP and PS1 phosphorylation in mammalian cells.** 293T cells were transfected with the indicated plasmids expressing APP (A), PS1 (C) and/or DYRK1A. Transfected cells were treated with the indicated doses of CX-4945 for 6 h, and then total cell extracts were subjected to western blot analysis. The phosphorylated and total APP and PS1 proteins from western blotting were quantified, and the amounts of protein relative to those of DMSO-treated samples are shown in B and D, respectively. The mean±s.d. values were determined from two independent assays. p-APP, phosphorylated APP; p-PS1, phosphorylated PS1; Vec, empty vector. / denotes the ratio of the two indicated proteins.

Next, we examined whether CX-4945 also inhibits DYRK1A in mammalian cells. The microtubule-associated protein Tau is a well-characterized substrate of DYRK1A, and aberrant phosphorylation of this protein is associated with the formation of NFTs in DS and AD (Tiraboschi et al., 2004). Therefore, we first investigated the effect of CX-4945 on Tau phosphorylation. In particular, phosphorylation of Tau at T212, which has been prominently observed in the brains of DYRK1A-overexpressing transgenic mice (Ryoo et al., 2007), was examined. Phosphorylation of Tau at T212 was induced by the overexpression of DYRK1A in 293T cells and then detected by western blotting with an antibody specific for phosphorylated Tau at Thr212. Under the same conditions, Tau phosphorylation was markedly decreased by treatment with CX-4945 in a dose-dependent manner with an IC_50_ of 100-200 nM (Fig. 3A and B). Moderate inhibition of Tau phosphorylation was seen at 0.1 μM, and almost complete inhibition was achieved at 1 μM. Importantly, the inhibitory efficacy of CX-4945 on DYRK1A was significantly stronger than that of harmine, INDY and proINDY (IC_50_ values of ∼500, 2000 and 2000 nM, respectively), which are well-known potent DYRK1A inhibitors (Ogawa et al., 2010; Smith et al., 2012) (Fig. S1). Similarly, CX-4945 strongly inhibited the phosphorylation of APP and PS1, which are also well-known substrates of DYRK1A and are crucial for amyloid plaque formation in DS and AD (Ryu et al., 2010; Smith et al., 2012), with estimated IC_50_ values of ∼80 and 100 nM for APP and PS1, respectively (Fig. 4). Collectively, these results clearly demonstrate that CX-4945 is a potent DYRK1A inhibitor *in vitro* and in mammalian cells.

### CX-4945 has a modulatory effect on DS- and AD-related calcineurin-NFAT signaling

**Fig. 5.**
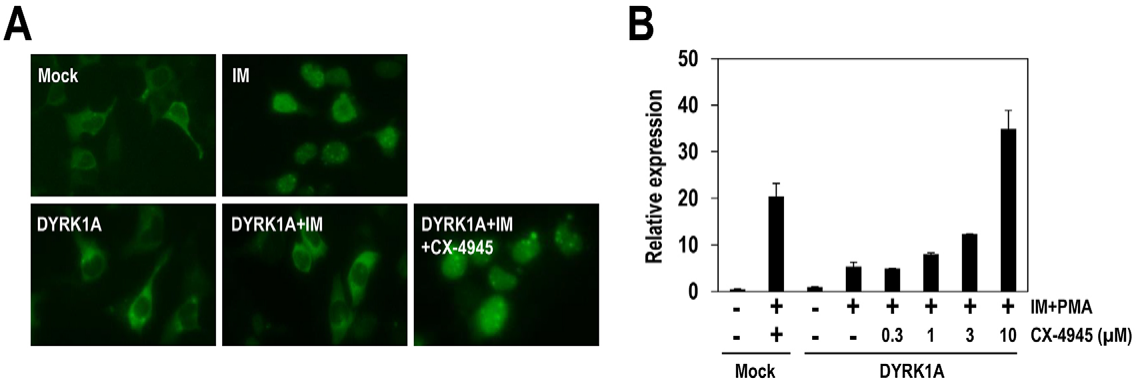
**CX-4945 has a modulatory effect on DYRK1A-associated NFAT signaling.** (A) The effect of CX-4945 on calcineurin-NFAT signaling was analyzed by visualizing the translocation of the NFATc1 protein. 293T cells were transfected with plasmids expressing Flag-NFATc1 or DYRK1A and then treated with CX-4945 (10 μM) or DMSO prior to stimulation with ionomycin (IM; 5 μM). (B) The effect of CX-4945 on NFATc1-mediated transcriptional activation. 293T cells were transfected with NRE-Luc reporter plasmid with or without plasmid expressing DYRK1A, and cells were then treated with IM (5 μM) and phorbol 12-myristate 13-acetate (PMA; 10 nM) along with the indicated doses of CX-4945. Firefly luciferase activities were measured using One-Glo reagents. Luciferase activity in the sample with reporter plasmid alone was set to 1, and the relative luciferase activities were calculated. Means±s.d. were determined from two independent experiments.

Because deregulation of calcineurin and nuclear factor of activated T cells (NFAT) signaling is related to the development of a DS- and AD-like phenotype, and because DYRK1A plays an important role in this signaling pathway (Arron et al., 2006), we examined the effect of CX-4945 on NFAT signaling involving DYRK1A by imaging the translocation of Flag-tagged NFATc1. Flag-NFATc1 remained predominantly in the cytosol when expressed in 293T cells (Mock, Fig. 5A). Upon the addition of the calcium ionophore ionomycin (IM), Flag-NFATc1 was translocated into the nucleus (Fig. 5A, IM) as a result of NFATc1 dephosphorylation by activated calcineurin. Overexpression of DYRK1A relocalized Flag-NFATc1 into the cytoplasm, even in the presence of ionomycin owing to the opposing action of DYRK1A on NFATc1 phosphorylation, which is consistent with its negative regulatory role in the calcineurin-NFAT pathway (Fig. 5A, DYRK1A+IM). Under these conditions, 10 μM of CX-4945 effectively induced the nuclear translocation of Flag-NFATc1, even in the presence of overexpressed DYRK1A (Fig. 5A, DYRK1A+IM+CX-4945).

The effect of CX-4945 on NFAT signaling was also examined with an alternative assay that measures NFATc1-mediated transcriptional activation, utilizing a firefly luciferase reporter driven by the NFAT response element (NRE-Luc). Treatment with IM and phorbol 12-myristate 13-acetate (PMA) induced a dramatic increase in luciferase activity (∼20 fold) as a result of the nuclear translocation of NFATc1 and the subsequent transcriptional activation (Fig. 5B). In this situation, the overexpression of DYRK1A suppressed the stimulatory effect of IM and PMA by 75%, resulting in a 4.5-fold increase in luciferase activity compared with that of the control (Fig. 5B). CX-4945 treatment reversed the effect of DYRK1A overexpression in a dose-dependent manner. The recovery effect was initiated at low micromolar concentrations (1 and 3 μM), exponentially increased at 10 μM of CX-4945 and eventually surpassed the luciferase activity from cells that lacked exogenous DYRK1A expression (Fig. 5B). The overriding effect on NFAT signaling seen at 10 μM of CX-4945 might be explained by the inhibition of endogenous DYRK-family kinases in 293T cells. These results further confirm the inhibitory function of CX-4945 on DYRK1A, particularly in DS- and AD-related signaling.

### CX-4945 restores neurological and phenotypic defects in a DS- and AD-like *Drosophila* model

*Drosophila melanogaster* is a highly tractable genetic model organism that can be used to investigate the molecular mechanisms of human diseases. Basic biological and neurological properties are highly conserved between humans and *Drosophila*, and nearly 75% of human disease-related genes, including *DRYK1A*, have a functional homolog in the *Drosophila* genome. Based on these advantages, *Drosophila* has been frequently utilized as a powerful *in vivo* model for the pharmacological validation of therapeutic drug candidates.

The *Drosophila* gene *minibrain* (*mnb*) is a well-studied ortholog of human *DYRK1A*. Similar to *DYRK1A* in humans, *mnb* is highly expressed in neural tissues in *Drosophila*, and mutations in *mnb* cause phenotypic defects in neuroblast proliferation and brain development. Similarly, the tissue-specific overexpression of *mnb* also induces various phenotypic and neurological defects in peripheral tissue, such as wings, as well as in the central nervous system (CNS) structure, recapitulating DS-like phenotypes in the *Drosophila* system (Degoutin et al., 2013).

In order to validate the pharmacological use of CX-4945 in an *in vivo* system, we established *mnb-*overexpressing flies and characterized the accompanying neurological and phenotypic defects. The most prominent phenotypic defect was observed in the morphology of the wing. Similar to in the report by Degoutin et al. (2013), wing-tissue-specific overexpression of *mnb* by the use of the *MS1096-gal4* driver caused incomplete formation of lateral vein 5 (LV5) with a penetration of 90% (Fig. S3). Surprisingly, feeding newly hatched larvae with CX-4945 for 2 weeks considerably improved the wing defect. This phenotypic rescue was seen when using each of the applied doses (1-25,000 nM) and was even apparent with nanomolar concentrations of CX-4945 (10-100 nM), whereas much higher concentrations of harmine (10-25 μM) were required to achieve a similar effect (Fig. S3). This result is consistent with our other observations that CX-4945 has stronger inhibitory effects compared to harmine (Figs 1 and 3; Figs S1-S3). Moreover, these results suggest that 100 nM of CX-4945 is an optimal concentration for further examination in an *mnb*-overexpressing *Drosophila* system.

**Fig. 6.**
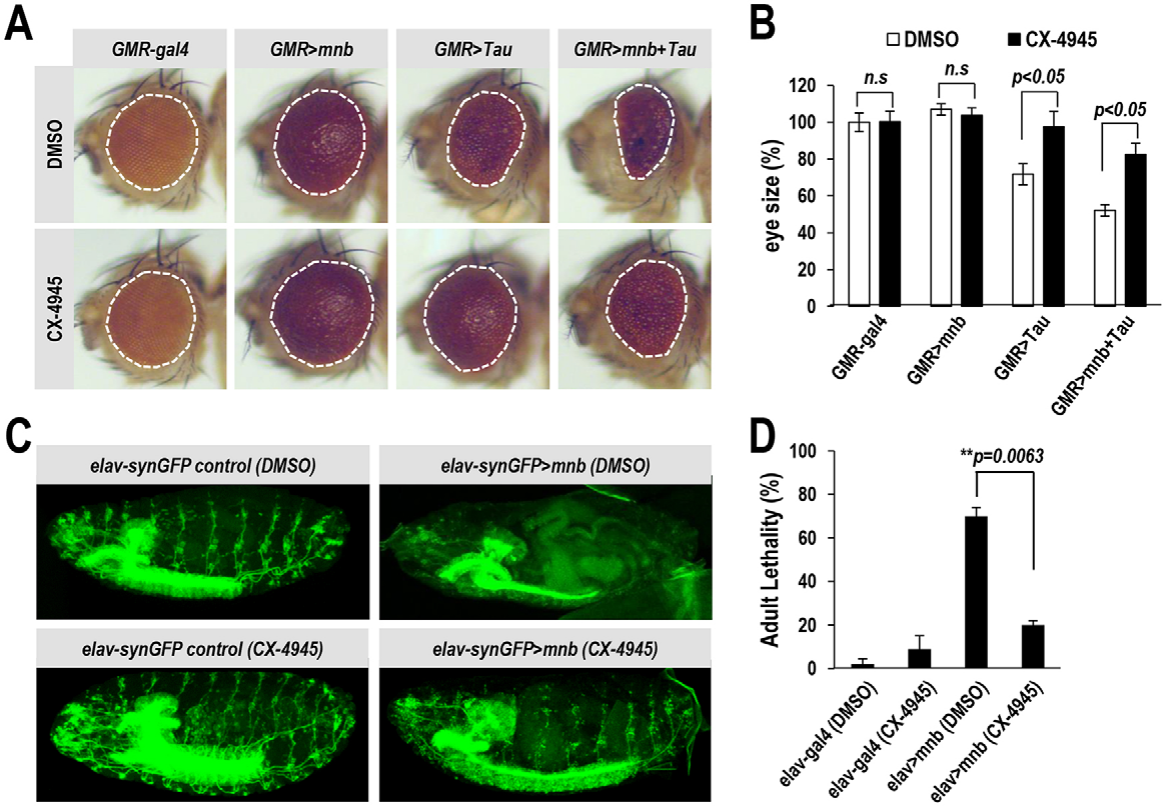
**CX-4945 rescues neurological and phenotypic defects in an *mnb*-overexpressing *Drosophila* model.** (A) The eyes of adult flies overexpressing *mnb* and/or human *Tau* under the control of the eye-specific *GMR-Gal4* driver, and of control flies bearing only *GMR-Gal4*. Overexpression of *mnb* aggravated the eye abnormality induced by *Tau* overexpression. This eye defect was effectively rescued by feeding with 100 nM of CX-4945. White dashed lines outline the eye contour. (B) The retinal surface areas were measured on multiple samples (*n*>10) from each genotype, and average eye sizes were presented as normalized percentages of the DMSO-treated *GMR-Gal4* control. (C) The brain cortex, primary neuronal cell clusters and axon bundle, as visualized with GFP (green) using *UAS-Synaptobrevin-GFP* driven by *elav-Gal4* at the late embryonic stage 18. The pattern of neural connectivity and CNS structure were severely disorganized in *mnb-*overexpressing embryos (*elav-synGFP>mnb*, DMSO), the neurogenic defects of which were remarkably rescued by 100 nM of CX-4945. (D) The pan-neuronal overexpression of *mnb* resulted in about 70% adult lethality. Feeding with 100 nM of CX-4945 dramatically reduced the lethality of the progeny by more than 70% (*n*=100 for each group). The *P*-value is depicted with an asterisk (**P<0.01) (two-tailed Student's *t*-test). Data are means±s.d. n.s., not significant.

As shown in Fig. 3A, Tau phosphorylation induced by DYRK1A overexpression was significantly decreased by CX-4945 in mammalian cells. Similarly, to test the pharmacological effects of CX-4945 on a Tau-mediated phenotype in the *Drosophila* model, *mnb* was overexpressed in eye tissue with or without the human Tau protein, using the UAS-Gal4 system. The expression of human Tau in *Drosophila* retinal tissue (*GMR>Tau*) produced a small rough eye phenotype (*GMR>Tau* in Fig. 6A and B), as described previously (Jackson et al., 2002). Coexpression of *mnb* enhanced Tau toxicity and resulted in a more severe phenotype with smaller eyes (*GMR*>*mnb+Tau* in Fig. 6A and B). Consistent with the inhibitory effect of CX-4945 on DYRK1A-mediated Tau phosphorylation in 293T cells (Fig. 3), eye defects induced by the overexpression of Tau alone (*GMR*>*Tau*) or with *mnb* (*GMR*>*mnb+Tau*) were significantly mitigated by feeding newly hatched larvae with 100 nM of CX-4945 [*GMR*>*Tau* (*CX-4945*) and *GMR*>*mnb+Tau* (*CX-4945*) in Fig. 6A and B]. Next, in order to analyze the effect of CX-4945 on the neurological phenotype, primary neuron clusters and their primary axon bundles were labeled with *UAS-Synaptobrevin-GFP*, driven by *elav-Gal4.* Pan-neuronal *mnb* overexpression (*elav::synGFP*>*mnb*) caused severe neurogenic defects in both the central and peripheral nervous system during embryogenesis [*elav::synGFP*>*mnb* (DMSO) in Fig. 6C]. Feeding parent flies with 100 nM of CX-4945 restored these neurogenic defects, particularly in the peripheral nervous system [*elav::synGFP*>*mnb* (*CX-4945*) in Fig. 6C]. It is noteworthy that CX-4945 itself had little noticeable effect on control flies that lacked *mnb* overexpression [*elav::synGFP*>*control* (*CX-4945*) in Fig. 6C]. Moreover, the viability of adult flies with the neuronal overexpression of *mnb* was examined. Pan-neuronal overexpression of *mnb* caused high rates of mortality in adult flies (∼70%, Fig. 6D). Surprisingly, feeding parent flies with 100 nM of CX-4945 dramatically reduced the mortality of the progeny by more than 70%. Collectively, these results indicate that CX-4945 can function as an inhibitor of DYRK1A/*mnb in vivo*, and can rescue the neurological and phenotypic defects of a DS- and AD-like *Drosophila* model.

### Oral administration of CX-4945 suppresses Tau hyperphosphorylation in the hippocampus of a DS-like mouse

**Fig. 7.**
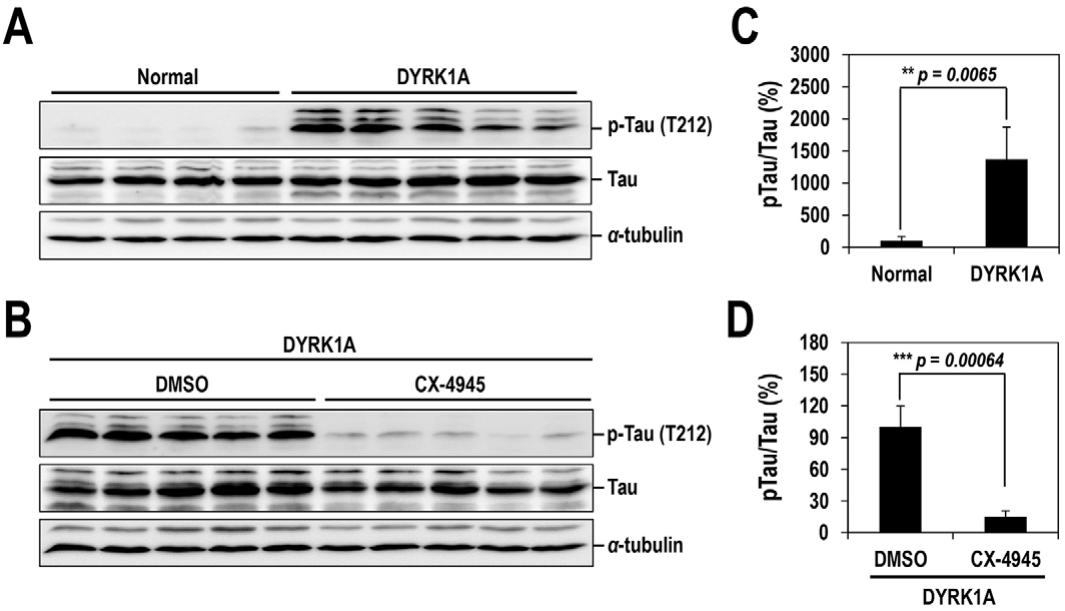
**Oral administration of CX-4945 suppresses Tau phosphorylation in DYRK1A-overexpressing mice.** (A) Hippocampuses from normal and DYRK1A-overexpressing C57BL/6 mice (*n*=5 for each) were harvested, and the phosphorylation of Tau was analyzed by western blotting with anti-phosphorylated-Tau (at residue T212; p-Tau) and anti-Tau antibodies. α-Tubulin was also analyzed as a loading control. (B) The phosphorylated and total Tau proteins in panel A were quantified, and the mean amount of each protein was calculated. Relative ratios of phosphorylated Tau to total Tau (pTau/Tau) were presented by setting the value from normal mice as 100%. (C) DYRK1A-overexpressing C57BL/6 mice (*n*=5 for each group) were administered orally with DMSO or 75 mg/kg of body weight of CX-4945 in PBS. Thirty minutes after oral administration, mice were killed, and the hippocampus was dissected from each mouse. Total cell lysates were prepared and subjected to western blotting with the indicated antibodies. α-Tubulin was also analyzed as a loading control. (D) The phosphorylated and total Tau proteins in panel C were quantified, and the mean amount of each protein across the animals was calculated. Relative ratios of phosphorylated Tau to total Tau were calculated by setting the value from DYRK1A mice that had been treated with DMSO as 100%. Data are means±s.d. (two-tailed Student's *t*-test).

In order to further validate the inhibition of DYRK1A by CX-4945 in a higher-level organism, mice with three copies of the DYRK1A gene, which mimics the trisomy seen in DS, were used. This mouse model has 50% higher expression of DYRK1A compared with a normal mouse, and increased Tau phosphorylation at T212 in the hippocampus has been well documented (Ryoo et al., 2007). Indeed, this phenomenon was also confirmed in our experiment. All DYRK1A-overexpressing mice exhibited higher levels of Tau phosphorylation than those seen in normal mice (Fig. 7A and B). Surprisingly, acute oral administration (30 min) of CX-4945 (75 mg/kg of body weight) remarkably decreased the phosphorylation of Tau (at T212), bringing it close to the levels seen in normal mice (Fig. 7C and D). This strong inhibitory effect of CX-4945 was still observed with samples harvested at 1 h after oral administration (data not shown). Collectively, these results indicate that CX-4945 efficiently penetrates the blood-brain barrier (BBB) and potently inhibits DYRK1A in the hippocampus of the DYRK1A transgenic mouse.

## DISCUSSION

DYRK1A has been suggested recently to be a promising molecular target for disease-modifying treatments for DS, especially in regards to the aspect of mental retardation. Even though a few compounds have been discovered and developed as potent inhibitors of DYRK1A, their therapeutic application is limited by pharmacological side effects. In this study, we identified a novel inhibitor of DYRK1A, CX-4945, which showed stronger inhibitory activity than harmine, INDY and proINDY, which are well-known potent inhibitors of DYRK1A. Moreover, CX-4945 significantly restored the neurological and phenotypic defects induced by the overexpression of *mnb*, an ortholog of human *DYRK1A*, in a *Drosophila* model, validating the pharmacological inhibition of DYRK1A by CX-4945 *in vivo*. Importantly, the DYRK1A inhibitory activity of CX-4945 was also proven in a DS-like mouse model through the dramatic suppression of Tau hyperphosphorylation.

Our extensive analysis using various assays clearly demonstrates that CX-4945 directly inhibits DYRKs with a high potency. First, CX-4945 inhibited all DYRKs tested in an *in vitro* kinase assay, showing the strongest inhibitory effect with DYRK1A and DYRK1B (IC_50_=∼6 nM for both, Fig. 1B). Using the same assay, the inhibitory activity of harmine (IC_50_=∼100 nM for DYRK1A), an alkaloid obtained from plants and a widely used inhibitor of DYRKs, was found to be about 20-fold weaker than that of CX-4945 (IC_50_=6.8 nM for DYRK1A) (compare Fig. 1B and C). Moreover, in a previous report, INDY and proINDY, which are benzothiazole derivatives identified as strong inhibitors of DYRK1A, showed slightly weaker inhibitory activities than harmine (Ogawa et al., 2010). Taken together, it appears that CX-4945 is more potent than harmine, INDY and proINDY. Second, stronger inhibition of DYRK1A by CX-4945 was also confirmed in mammalian cells through the analysis of DYRK1A-mediated Tau phosphorylation. The estimated IC_50_ of CX-4945 (∼100-200 nM) on Tau phosphorylation that had been induced by DYRK1A overexpression was lower than that of harmine, INDY and proINDY (∼500, 2000 and 2000 nM, respectively; compare Fig. 3 and Fig. S1). Third, NFATc1-dependent transcriptional activation in calcineurin-NFAT signaling suppressed by the overexpression of DYRK1A was dose-dependently derepressed by treatment with CX-4945, harmine, INDY and proINDY. However, CX-4945 exhibited a stronger effect than harmine at the same concentration (Fig. S2). Fourth, a stronger effect of CX-4945 was observed in phenotype-rescue experiments using an *mnb*-overexpressing *Drosophila* model (Fig. S3). Notably, the oral administration of CX-4945 dramatically suppressed the phosphorylation of Tau in the hippocampus of DS-like mice within 30 min (Fig. 7), demonstrating its efficient penetrability through the BBB and DYRK1A-inhibitory activity in a higher-animal model. Collectively, these results firmly demonstrate that CX-4945 is a potent inhibitor of DYRK1A *in vitro*, in mammalian cells and in living organisms.

Intriguingly, the chemical structure of CX-4945 is quite similar to that of harmine, except that CX-4945 has an additional substructure appended to the central tricycle (Fig. S4). It is conceivable that this appendage might contribute considerably to the enhanced inhibitory activity of CX-4945, considering that the inhibitory activity of CX-4945 is about threefold stronger than that of harmine in mammalian cells and 20-fold stronger *in vitro* (Figs 1 and 3). INDY and proINDY also have some degree of similarity to the core structure of CX-4945 (Fig. S4). Consistent with the similarity in chemical structures, the predicted structure of the CX-4945-DYRK1A complex is quite similar to co-crystal structures of the harmine- and INDY-DYRK1A complexes. Determination of the co-crystal structure of CX-4945-DYRK1A in a future study would improve our understanding of the molecular basis of inhibition.

Including harmine, INDY and proINDY, currently available DYRK1A inhibitors can be classified into two categories: natural and synthetic products. Harmine and epigallocatechin gallate (EGCG) are well-studied natural compounds (Smith et al., 2012). INDY, proINDY, roscovitine, purvalanol A, pyrazolidine-diones, amino-quinazolines, meridianins, pyridine and pyrazines, and chromenoidoles are all synthetic compounds with varying potencies towards DYRK1A (Smith et al., 2012). Among these compounds, harmine, INDY and proINDY have drawn particular attention because of their high potency and selectivity. Harmine is currently considered to be a potent inhibitor, but behavioral side effects related to monoamine oxidase A (MOA) inhibition and hallucinogenic stimulation in animal models have limited its therapeutic application (Kim et al., 1997). According to a report by Ogawa et al. (2010), INDY and its acetylated analogue proINDY are a little less potent but more selective than harmine. Importantly, the *in vivo* use of proINDY had been partly validated by showing the considerable rescue of neurological and morphological defects induced by the overexpression of *Xenopus* DYRK1A during development of *Xenopus* embryos. However, further extensive pharmacological validation in higher-level organisms is required. In addition, EGCG, a well-known phenolic compound from green tea, is of particular interest given that beneficial effects on cognitive defects in a DS mouse has been observed (De la Torre et al., 2014). Even though EGCG is the sole compound that has been pharmacologically validated in an animal model, the nonselective nature and low potency of EGCG remains to be tested further in humans in a clinical setting. Extensive evaluation of leading or the most advanced candidate compounds suggests that undesirable side effects are the limiting factors for their therapeutic application. In that sense, CX-4945, a newly identified inhibitor of DYRK1A, would hold promise because its safety has been already proven in phase I clinical trials for use as a cancer therapeutic. To our knowledge, CX-4945 is the only drug candidate under clinical investigation for which the DYRK1A-inhibitory effect has been proven. Currently, phase 1b and phase 2 studies are underway in combination with gemcitabine and cisplatin for the frontline treatment of individuals with bile duct cancers (cholangiocarcinoma), according to a release from Senhwa Biosciences (2014).

In addition to its significance in DS, the overexpression or hyperactivity of DYRK1A has been also reported in the brains of individuals diagnosed with any one of several neurodegenerative diseases, including AD, Parkinson's, Huntington's and Pick's (Ferrer et al., 2005; Kang et al., 2005; Kim et al., 2006). In particular, the central role of DYRK1A in the pathogenesis of AD has been well documented in a growing body of evidence. First, most DS individuals over 30 years old develop amyloid plaques and NFTs, which are the typical neuropathologies observed in the brains of individuals with AD, leading to the progressive neurodegeneration and high incidences of dementia at older ages (Dierssen and de Lagran, 2006; Wegiel et al., 2011). Second, DYRK1A directly phosphorylates Tau as well as APP and PS1, and aberrant and/or excessive phosphorylation of these targets is believed to be crucial for the formation of NFTs and amyloid plaques, respectively (Ryoo et al., 2008, 2007; Ryu et al., 2010). These observations provide the molecular link between DS and AD, and further suggest that DYRK1A is a promising therapeutic target for treating diseases such as AD and DS. In this regard, CX-4945, with a high potency and proven safety in a clinical setting, will be a useful tool in DYRK1A-related research and can serve as a drug candidate for the treatment of DS and AD.

In addition to neurodegenerative diseases, DYRK1A has also been reported to be associated with cancers and metabolic diseases. DYRK1A is involved in the progression of cancer by modulating several pathways related to proliferation and migration (Ionescu et al., 2012). The involvement of DYRK1A in metabolic events has been demonstrated particularly by our study with *mnb*, an ortholog of human DYRK1A, in the *Drosophila* model. Overexpression of *mnb* in peptidergic neurons results in increased food intake (Hong et al., 2012). Notably, a recent study has highlighted the significance and versatility of DYRK1A by showing that inhibition of DYRK1A by harmine increases human pancreatic β-cell proliferation through the modulation of NFAT signaling (Wang et al., 2015), indicating a new therapeutic approach for diabetes. Therefore, targeting DYRK1A would be broadly applicable as a therapeutic approach towards at least some types of cancers and metabolic diseases.

Our research suggests that CX-4945, a drug candidate for cancer treatment, can be repurposed for disease-modifying treatment of DS and AD. Further pharmacological validation in higher-animal models for DS and AD is required in future studies.

## MATERIALS AND METHODS

### Cell culture, drug treatment and transfection

293T cells were cultured in Dulbecco's modified Eagle's medium (DMEM) containing 10% fetal bovine serum (FBS; Hyclone) supplemented with 1% penicillin and streptomycin. CX-4945 (Selleckchem), harmine (Sigma), INDY and proINDY (Tocris) were dissolved in dimethyl sulfoxide (DMSO) prior to treatment. Cells were seeded at approximately 50% confluence into 6-well cell culture plates, maintained overnight and then transfected with the appropriate plasmids using the XtremeGene Transfection Reagent (Roche) according to the manufacturer's instructions.

### Plasmid construction

PcDNA3.1-DYRK1A, -Tau, -APP and -C-PS1 were constructed as previously described (Ryoo et al., 2008, 2007; Ryu et al., 2010). To generate a plasmid expressing the Flag­-NFATc1 protein, *NFATC1* cDNA was amplified by using PCR with appropriate primers, and the DNA fragment was inserted into the EcoRI site of the pcDNA3.1-Flag plasmid.

### Quantitative western blot analysis

Quantitative western blot analysis was performed as described previously (Choi et al., 2014). The following antibodies were used: anti-Tau antibody (HT7, catalog # MN1000, ThermoFisher Scientific, 1:1000 dilution), anti-phosphorylated-Tau (at residue T212) antibody (catalog # 44740G, Invitrogen, 1:1000 dilution), anti-glyceraldehyde-phosphate-dehydrogenase (GAPDH) antibody (G-9, catalog # sc-365062, Santa Cruz Biotechnology, 1:2000 dilution), anti-PS1 antibody, (catalog # 3622, Cell Signaling Technology) as well as anti-APP, anti-phospho-APP (at residue T668) and anti-Flag antibodies (catalog # 2452, catalog # 2451, catalog # 9146, all Cell Signaling Technology, 1:1000, 1:1000 and 1:1000 dilution, respectively). Anti-DYRK1A antibody (1:500 dilution) was generated as described previously (Ryoo et al., 2007). Anti-hnRNP-A1 antibody (1:1000 dilution) was kindly provided by Gideon Dreyfuss (University of Pennsylvania, PA).

### *In vitro* kinase assays

Kinase assays were conducted using the Kinase Profiler services offered by Life Technologies, which utilizes a fluorescence-based immunoassay. Detailed protocols can be found at http://www.lifetechnologies.com/kinaseprofiling. The inhibitory activity of each kinase (DYRK1A, DYRK1B, DYRK3 and DYRK4) was measured with five concentrations of CX-4945 over a range of 0.001 to 10 μM, and the IC_50_ was determined using the GraphPad Prism 5 software. To determine whether CX-4945 acts by competing with ATP for the inhibition of DYRK1A, kinase activity was measured in the presence of various concentrations of ATP (50, 75, 100, 150 and 200 μM), and IC_50_ values were determined using the GraphPad Prism 5 software. All experiments were performed twice.

### Computer-aided molecular docking

To build a structural model of DYRK1A in complex with CX-4945, we performed molecular docking studies by using the CDOCKER program (Wu et al., 2003) in Discovery Studio 3.1 software (Accelrys). The CX-4945 ligand was generated by using ChemBioDraw, and the energy-minimized structure was transferred to Discovery Studio 3.1. The crystal structure of DYRK1A obtained from the RCSB Protein Data Bank (PDB code: 3ANQ) was used for docking studies. Ten initial random conformations were taken, and ten initial poses were chosen for final refinement. CHARMm forcefield was employed for molecular dynamics and dynamics steps, and target temperatures were set to be 1000 and 1000 K, respectively. The structure with the lowest CDOCKER interaction energy was selected to be a final structure. Figures of the complex were drawn by using the PyMOL software package.

### NFATc1 localization by immunofluorescence

293T cells were seeded into 8-well chambered coverslips (Ibidi) and transfected with a plasmid expressing Flag-NFATc1. On the next day, cells were pre-treated with CX-4945 (10 μM) for 3 h and then stimulated with IM (5 μM) for 1 h. Cells were fixed with 4% paraformaldehyde in PBS for 10 min at room temperature, followed by permeabilization with 0.1% Triton X-100, and then subjected to immunofluorescence staining with anti-Flag (1:500) antibody for 2 h. Cells were then washed with cold PBS three times for 3 min each, incubated with Alexa-Fluor-488-conjugated anti-rabbit IgG (1:800) (Invitrogen) at room temperature for 1 h and then examined under a fluorescence microscope.

### NFAT-RE promoter assay

The firefly luciferase reporter assay was performed using a One-Glo Assay System (Promega) according to the manufacturer's instructions. The transcriptional activity of NFATc1 in 293T cells was measured with a reporter vector (pGL4.30 luc2P/NFAT-RE/Hygro) (Promega) that expresses the firefly luciferase gene under the control of an NFAT-RE promoter. 293T cells were transfected with the DYRK1A-expressing plasmid and reporter plasmid. On the next day, cells were treated with IM (2.5 μM) and phorbol-12-myristate-13-acetate (10 nM) along with the indicated amounts of inhibitors. Cells were further incubated for 8 h and then cell lysates were harvested to measure luciferase activity.

### *Drosophila* culture and drug administration

*Drosophila melanogaster* were cultured at 25°C on standard cornmeal medium. *GMR-gal4* (#9146), *MS1096-gal4* (#8860), *elav*[*C155*]*-gal4::UAS-synGFP* (#6923), *UAS-Tau* (human wild-type Tau, #51362) and all other stocks and balancers were obtained from the Bloomington Stock Center (Bloomington, IN). *UAS-mnb* transgenic flies have been described in our previous reports (Hong et al., 2012); the full-length coding sequence of *Drosophila mnb* (CG 42273) was subcloned into the pUAS vector, and *UAS-mnb* transgenic flies were obtained by using the P-element-mediated germline transformation. For defining optimal concentration of chemical inhibitors, 50 embryos of each genotype were raised on drug-containing food (0, 1 nM, 10 nM, 100 nM, 1 µM, 10 µM and 25 µM in 0.05% DMSO) and allowed to develop to adulthood (viability assay and wing vein quantification assay). Experiments were repeated at least three times. For analyzing the eye phenotype, newly eclosed flies were collected and allowed to mate for 2-3 days and then transferred to medium containing 100 nM CX-4945 or DMSO as a control. Flies were exposed to the drug until adult stage, and the eye phenotypes of newly eclosed male were photographed using the Olympus SZ60 binocular microscope equipped with an eXcope K5 CCD system. Fly eye sizes were measured on multiple samples (*n*>10) from each genotype using the National Institutes of Health ImageJ software. Average eye size was presented as a normalized percentage of control eye size.

For analyzing the embryonic neurological phenotypes and lethality, newly eclosed virgin females or male flies were raised on standard medium containing 0.5% DMSO or 100 nM CX-4945 for 7 days before mating. CX4945-exposed virgins and males were allowed to mate in drug-containing medium. Embryos from CX-4945-fed parent flies were collected and staged at 25°C. They were dechorionated and fixed, and immunostaining was performed as described previously (Lee et al., 2008). The samples were then rinsed and washed three times for 15 min each time in PBT (PBS, 0.1% Triton X-100) and blocked for 1 h in PBT plus 10% normal goat serum. Primary antibodies were incubated overnight at 4°C. Then, samples were washed and incubated in secondary antibodies for 2 h at room temperature, washed again and mounted in Vectashield (Vector Laboratories, Burlingame, CA). The primary antibody used was chicken anti-GFP (Abcam, catalog #ab13970, 1:10,000), and Alexa-Fluor-488-coupled secondary antibodies were from Molecular Probes. Embryonic neurological phenotypes were imaged on an Olympus FV1000 confocal microscope (Olympus, Center Valley, PA) and processed using ImageJ. For adult lethality recordings, at least 100 flies were used for each individual experiment. For paired samples, two-tailed Student's *t*-test was used to analyze the effects of CX-4945 on eye phenotype and adult lethality.

### Tau phosphorylation in the hippocampus of DYRK1A TG mouse

DYRK1A transgenic mice with three copies of the human *DYRK1A* gene were maintained as described previously (Ahn et al., 2006). Experiments were performed in accordance with the guidelines under the approval of the Institutional Review Committee for Animal Care and Use, KRIBB, Daejeon, Korea. The 8- to 10-week-old C57BL/6 mice were administered with DMSO or CX-4945 (75 mg/kg of body weight) orally in PBS solution, and the hippocampus was dissected after 30 min. The hippocampal lysates were prepared by using a Digital Sonifier Cell Disruptor instrument (BRANSON) in CelLytic MT buffer (catalog # C3228, Sigma-Aldrich) containing protease inhibitor cocktail set III (catalog # 535140-1ML, Calbiochem). The protein concentration was determined using the Bradford protein assay (Bio-Rad). Proteins were separated by SDS-PAGE and analyzed by immunoblotting with antibodies against phosphorylated Tau and Tau using an LAS4000 image analyzer (Fujifilm, Tokyo, Japan).

### Statistical analysis

Data were analyzed by two-tailed paired or unpaired Student's *t*-tests. *, ** and *** represent *P*<0.05, *P*<0.01 and *P*<0.001, respectively, which are considered statistically significant.
